# Scarlet Fever in the Tropics

**Published:** 1899-09-16

**Authors:** 


### SCARLET FEVER IN THE TROPICS.

Several articles have appeared lately in the *Journal of Tropical Medicine* in regard to the rarity with which some common diseases of temperate climates are met with in the tropics. The prevalence of scarlet fever, for example, is stated to diminish as the equator is approached, whether from the north or the south. Dr. Kenneth Macleod summarises the matter in regard to India as follows: Scarlet fever has been frequently imported by troop ships, and small epidemics have thus arisen in Indian natives, especially among children. These epidemics have always been of a limited kind, and have speedily died out. Such epidemics appear to have been more substantial and protracted in the hill stations than on the plains. Cases and groups of cases have been observed the origin of which could not be ascertained. The disease does not appear to prevail epidemically among indigenous races and populations. Cases of an exanthem closely resembling, if not identical with, scarlet fever, have been observed in Calcutta and elsewhere, but these have mostly been single or limited to one family, and have manifested no disposition to become diffused. The following totals from the reports published by the Sanitary Commissioner with the Government of India for the 20 years, 1877 to 1896, may be quoted—European troops (strength about 60,000): Cases of scarlet fever, 56, deaths, 2; women (strength about 4,000), cases, 7, deaths, 0; children (strength about 7,000), cases, 124, deaths, 10. Native troops (strength about 125,000): Cases, 20, deaths, 1; prisoners (strength about 110,000), no cases.

